# Effectiveness of blood volume change–guided ultrafiltration control (BV-UFC) in hemodialysis: a crossover comparative study

**DOI:** 10.1093/ckj/sfaf141

**Published:** 2025-05-06

**Authors:** Shintaro Hamada, Masami Hata, Shuntaro Furukawa, Sunao Yamamoto

**Affiliations:** Department of Nephrology, Sanin Rosai Hospital; Department of Clinical Engineer, Sanin Rosai Hospital; Department of Clinical Engineer, Sanin Rosai Hospital; Department of Nephrology, Sanin Rosai Hospital

**Keywords:** blood volume change-guided ultrafiltration control, hemodialysis, intradialytic hypotension, plasma refilling

## Abstract

**Background and hypothesis:**

Intradialytic hypotension (IDH), a common hemodialysis (HD) complication, increases cardiovascular risks and affects prognosis. Rapid ultrafiltration (UF) is a key factor. The blood volume change–guided ultrafiltration control (BV-UFC) system, which adjusts UF rates based on real-time blood volume (BV) monitoring, aims to enhance hemodynamic stability and reduce IDH.

**Methods:**

A 6-week crossover trial compared BV-UFC and standard HD in reducing IDH. Patients underwent 2 weeks of each treatment. The primary outcome was the frequency of IDH episodes. Secondary outcomes were plasma refilling rate (PRR) and target UF volume achievement.

**Results:**

This study included 38 patients, with 31 patients completing the trial. The frequency of IDH episodes was significantly reduced in patients using the BV-UFC system (*P* = .019). HD sessions with BV-UFC system showed a significantly higher PRR throughout the treatment session, particularly during the first 0–1 h and 1–2 h of treatment (*P* = .019, *P* < .001, *P* < .001), as compared with standard HD and the target UF volume was consistently higher in the BV-UFC sessions (*P* < .01).

**Conclusion:**

The BV-UFC system effectively reduced the incidence of IDH by automatically adjusting the UF rate based on BV, without compromising treatment safety or UF volume. These findings suggest that BV-UFC can enhance hemodynamic stability and improve dialysis outcomes in patients undergoing HD (jRCT Study No. jRCT1062230034, registration date: 1 July 2023).

## Graphical Abstract

**Graphical Abstract:**
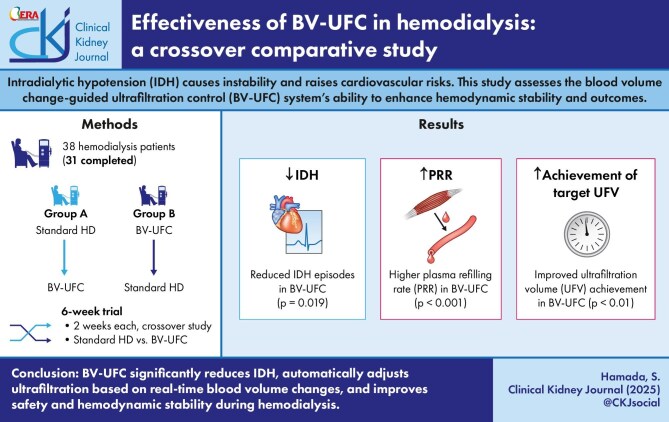

KEY LEARNING POINTS **What was known:**

- Intradialytic hypotension (IDH) is a frequent complication during hemodialysis, leading to cardiovascular risks.
- Blood volume (BV) monitoring has shown potential in improving hemodynamic stability by adjusting ultrafiltration rates, but the effectiveness of BV change–guided ultrafiltration control (BV-UFC) systems had not been conclusively proven.

**This study adds:**

- This study demonstrates that BV-UFC significantly reduces the incidence of IDH by automatically adjusting the ultrafiltration rate in response to real-time BV changes, without compromising treatment safety.
- Hemodialysis sessions with BV-UFC also show improved plasma refilling rates and target ultrafiltration volume achievement.

**Potential impact:**

- The BV-UFC system enhances hemodynamic stability during hemodialysis, potentially reducing the risk of cardiovascular events associated with IDH.
- This technology could improve dialysis outcomes and patient safety, paving the way for broader adoption in clinical practice to manage fluid balance more effectively.

## INTRODUCTION

Intradialytic hypotension (IDH) presents a substantial challenge in the management of patients undergoing hemodialysis (HD) [1]. This complication, characterized by a critical drop in blood pressure (BP) during dialysis sessions, not only compromises the efficiency of the HD procedure but also poses serious risks of cardiovascular events and long-term outcomes [2–4]. An increased risk of IDH is associated with several patient-related characteristics and comorbidities including advanced age, diabetes mellitus, ischemic heart disease, left ventricular hypertrophy and autonomic dysfunction [5–7]. These factors contribute to heightened susceptibility to IDH by disrupting compensatory mechanisms, thereby impairing cardiovascular stability during HD. Rapid ultrafiltration (UF) rates have been shown to negatively impact survival outcomes [8], whereas fluid overload is associated with poor prognosis [9, 10]. Excessive UF during HD often causes IDH due to insufficient plasma refilling from the interstitial to capillary circulation. Rapid fluid removal exceeds the vascular system's capacity to replenish plasma, leading to reduced blood volume (BV), hypotension, and compromised hemodynamic stability, which can worsen patient morbidity [11, 12]. Prolonged dialysis has been shown to improve the prognosis by enabling adequate UF and lowering the UF rate [13]. However, this approach imposes a greater burden on the HD patients and staff. A key challenge in HD therapy is achieving the same UF volume within the same treatment timeframe, while avoiding IDH and improving patient outcomes.

Recent advancements in dialysis technology have enabled non-invasive and highly accurate monitoring of BV in HD devices [14–16]. Continuous BV monitoring has been reported as a potential method for early detection of hemodynamic changes associated with IDH [17, 18]. Using an intradialytic BV monitoring (IBVM) device, UF rates were manually adjusted based on BV monitoring during the CLIMB trial. However, this approach was associated with increased hospitalization and mortality rates compared with standardized monitoring [19]. These findings suggest that manually adjusting UF rates based on BV monitoring presents significant challenges for dialysis staff and may not effectively mitigate adverse outcomes. In contrast, dialysis devices by Nikkiso Co., Ltd (Tokyo, Japan) feature a BV change–guided ultrafiltration control (BV-UFC) system that adjusts automatically the UF rate every 2 min based on BV measurements, following a target BV reduction curve. This system theoretically aligns UF rates with plasma refilling, reducing IDH incidence. However, a previous crossover trial showed no significant difference in IDH incidence between standard HD and HD with the BV-UFC system [20]. A previous study used a BV-UFC system with near-default settings, though these are not always straightforward to use. In this study, we conducted a crossover trial to examine hemodynamic responses during HD after modifying BV-UFC settings to simplify configuration.

## MATERIALS AND METHODS

### Subjects

Outpatients undergoing HD in our hospital who met the following criteria were enrolled: (i) 20 years  ≤ age ≤ 90 years; (ii) can get a blood flow rate of 200 mL/min or more by vascular access; and (iii) have been receiving HD for at least 3 months before study enrollment. Pregnancy and vascular access recirculation were excluded. The patients were randomly assigned into two groups in an open-label design with R (The R Foundation for Statistical Computing, Vienna, Austria).

### Study design

We conducted a 6-week crossover intervention study to observe hemodynamics in patients undergoing HD. The study consisted of a 2-week run-in period, followed by a 2-week standard HD, and then another 2-week HD with BV-UFC. A multipurpose dialysis-monitoring device DCS-200Si (Nikkiso, Tokyo, Japan) was used throughout all study periods. Patient recruitment was performed from 1 June 2023, to 1 February 2024. This study was conducted between 1 August 2023, and 30 May 2024.

### Definitions

BP was measured at the start of HD and every 20 min using an electronic monitor. IDH was defined as a drop in systolic BP of ≥20 mmHg or mean arterial pressure (MAP) of ≥10 mmHg from baseline [21].

Treatments for symptomatic IDH (e.g. nausea, dizziness, cramps) included adjusting the bed to the Trendelenburg position, modifying UF rate (standard HD only), administering oral peony licorice solution and saline infusion.

### Run-in period

**Figure 1:**
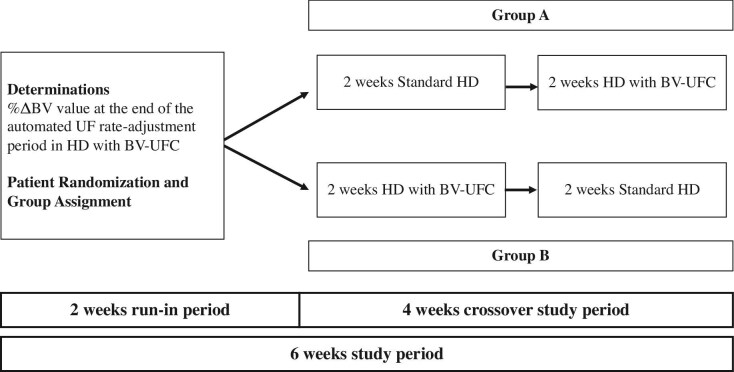
Flow diagram of the study. This figure illustrates the study design, including the 6-week study period, initial 2-week run-in phase, and subsequent 4-week crossover period. Patient randomization and group assignments for standard HD and HD with BV-UFC are shown.

The dry weight in each facility was determined using various methods, including physical assessments, hemodynamic status during HD, cardiothoracic ratio measured by chest X-ray, post-HD human atrial natriuretic peptide levels, and the degree of %ΔBV reduction by a multidisciplinary team consisting of nephrologists, nurses and clinical engineers. To appropriately achieve dry weight in HD with BV-UFC, BV-UFC settings were established during a 2-week run-in period for each enrolled patient and remained unchanged throughout the crossover study period. During this 2-week period, the lower limit alarm for systolic BP was set between 110 and 130 mmHg for each patient, as is customary in regular dialysis practice (Fig. 1).

### UF rate setting during HD with the BV-UFC system

HD with BV-UFC was divided into four phases based on changes in the UF rate. The default settings were as follows: (i) constant UF rate for the first 10 min, calculated as body weight gain divided by HD time (constant UF rate period); (ii) 1.3 × higher UF rate for the next 60 min (higher UF rate period); (iii) automatic UF adjustments based on ΔBV every 2 min until 20 min before HD completion, targeting reductions of 50%, 80% and 95% (automatic UF rate–adjustment period); and (iv) constant UF rate in the final 20 min to reach dry weight (automatic constant UF rate period). Adjustments were made to time intervals, UF rates and target %ΔBV; however, default settings had issues, including excessively high UF rates if patients exceeded dry weight, challenging ΔBV targets and large UF rates at the end. To address this, we shortened the second phase to 10 min with a 1.0 × UF rate, set the target %ΔBV of the third phase to 2%–4% higher than that at the end of standard HD in the third phase, and reduced the UF rate to 50% of the remaining UF volume for the final 20 min in the fourth phase. The length of the third phase was determined by subtracting the total duration of the first, second, and fourth phases (a total of 40 min) from the total dialysis time. For example, if the total dialysis time is 240 min, the third part corresponds to 200 min (Table 1).

**Table 1:** BV-UFC settings.

	BV-UFC settings			
	Default settings		This study	
	Times (min)	UF rate (%)	Times (min)	UF rate (%)
Constant UF rate period (1st part)	10	100	10	100
Higher UF rate period (2nd part)	60	130	10	100
Automatic UF rate adjustment period (3rd part)	HD time: 90 min		HD time: 40 min	
Automatic constant UF rate period (4th part)	20	100	20	50

This table compares the default settings of the BV-UFC system with the settings used in this study. The time duration and UF rate for each phase—constant UF rate period (1st part), higher UF rate period (2nd part), automatic UF rate adjustment period (3rd part) and automatic constant UF rate period (4th part)—are indicated.

### Crossover study period

During this period, patients in Group A underwent standard HD for 2 weeks, followed by 2 weeks of HD with BV-UFC. Group B patients underwent HD with BV-UFC for 2 weeks, followed by 2 weeks of standard HD. All participants in this study underwent HD three times per week. This crossover comparative study included a total of 12 HD sessions for each participant: 6 sessions over 2 weeks with standard HD and 6 sessions over 2 weeks with BV-UFC-guided HD. During the 4-week period, each patient underwent a crossover between standard HD and HD with BV-UFC, with no changes in dry weight, antihypertensive medication or BV-UFC settings. Data from 12 HD sessions over a 4-week period were collected.

### Primary outcome

The primary outcome was differences in the number of IDH episodes between the two conditions.

The number of IDH episodes per HD session was evaluated using BP measurements every 20 min.

### Secondary outcomes

(i) The average plasma refilling rate (PRR)

The average plasma refilling rate was evaluated to assess hemodynamics during HD.

PRR was calculated using the following formula:


\begin{equation*}
{\mathrm{PRR}} = {\mathrm{\ }}\frac{{{\mathrm{UF\ volume\ during\ the\ measurement\ period}} + {\mathrm{\Delta BV}}\left( {{\mathrm{increase\ in\ blood\ volume\ during\ the\ measurement\ period}}} \right)}}{{{\mathrm{Duration\ of\ the\ measurement\ period}}}}
\end{equation*}

### Detailed explanation

UF volume during the measurement period: the ultrafiltration volume (in mL) removed by the dialysis machine during the measurement period; ΔBV(increase in BV during the measurement period): calculated using the ΔBV change rate during the measurement period; duration of the measurement period: each measurement period was fixed at 60 s (1 min).

The initial BV at the start of each dialysis session is automatically estimated using pre-dialysis body weight (kg)/13. The average PRR (L/h) was recorded every 20 s using the DCS-200Si device during the entire dialysis session. The values were adjusted for each patient's dry weight (kg) to calculate the average PRR (mL/kg/h). PRR values were then averaged for the following intervals: the entire HD session, 0–1 h, 1–2 h, 2–3 h and 3–4 h after dialysis initiation.

The PRR values during the first 16 min after HD initiation were excluded from the analysis because of potential instability caused by pre-HD walking and infusion of priming saline into the body.

(ii) Achievement rate of target UFV

Achievement of the target ultrafiltration volume (UFV) at the end of HD was defined as being within ± 0.3 kg of the dry weight.

(iii) Number of treatment episodes for IDH

The frequency of operations and interventions performed by the medical staff during HD included rapid adjustment of the dialysis bed to the Trendelenburg position, modification of the UF rate (in standard HD only), saline infusion, and lower limit alarms for systolic BP.

### Statistical analyses

Data on patients’ general characteristics are presented as mean ± SD, calculated from the averages of all patients included in the study. Clinical data from standard HD and HD with BV-UFC are presented as mean ± standard error, based on the mean values of all hemodialysis sessions conducted during the study period for each patient. This approach ensures that the results reflect overall trends across sessions rather than individual session variability. Paired t-tests were used to compare clinical parameters between HD and HD patients with BV-UFC. The chi-square test was used to the number of dialysis sessions which at least one hypotensive event (systolic blood pressure decrease more than 20 mmHg). McNemar's test was used to evaluate the achievement rate of the target UF volume at the end of HD. All analyses were performed using StatFlex ver7 (Artech Co., Osaka, Japan). Statistical significance was set at *P* < .05. The 95% confidence intervals (CI) were calculated using the model parameter coefficients and standard error (SE).

This study did not apply an intention-to-treat (ITT) analysis, as seven participants withdrew before receiving any treatment (five did not start the crossover trial, and two discontinued before initiating either standard HD or BV-UFC). Therefore, a per-protocol analysis was performed, including only participants who completed the trial.

### Ethical approval

This study was registered in the Japan Registry of Clinical Trials (jRCT Study No. jRCT1062230034, registration date: 1 July 2023) and was approved by the Ethics Committee of Sanin Rosai Hospital (approval number: 11 000 999). The study was conducted in accordance with the principles outlined in the Declaration of Helsinki (Fortaleza, 2013). Informed consent was obtained from all participants prior to their enrollment in the study.

In the initial study design, PRR was selected as the primary endpoint based on prior study suggesting that the default settings of the BV-UFC system would have minimal impact on IDH frequency [20]. However, during the preparatory phase, modifications to the BV-UFC settings led to a significant reduction in IDH frequency. Based on recommendations from peer reviewers, the study endpoints were revised. The primary endpoint was changed from PRR to the frequency of IDH, and PRR was designated as a secondary endpoint. This revision was approved by the ethics committee before implementation.

## RESULTS

### Baseline characteristics

**Figure 2:**
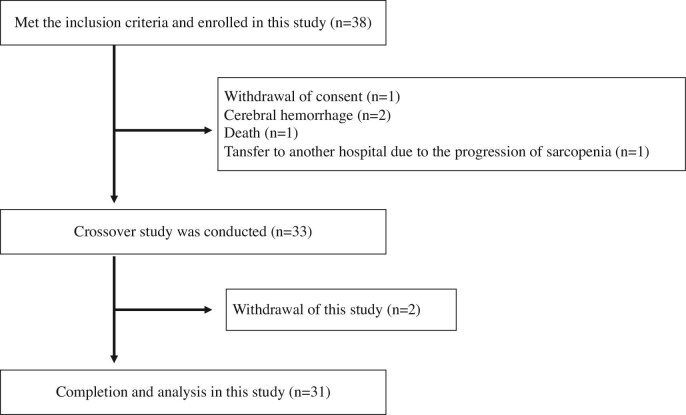
Patient flow chart. This chart illustrates the progression of patients through the study. Out of 38 enrolled participants, 5 withdrew before the crossover phase due to consent withdrawal (*n* = 1), cerebral hemorrhage (*n* = 2), death (*n* = 1) and sarcopenia-related hospital transfer (*n* = 1). The remaining 33 patients entered the crossover phase, with two additional withdrawals due to complications. A total of 31 patients completed the study and were included in the final analysis.

A total of 38 patients were enrolled in the study, but 5 were excluded before implementation due to consent withdrawal (*n* = 1), cerebral hemorrhage (*n* = 2), death (*n* = 1) and sarcopenia-related hospital visit difficulties (*n* = 1). Two more patients did not complete the study due to sarcopenia-related dry weight adjustments (*n* = 1) and acute myocardial infarction (*n* = 1) (Fig. 2). Among the 31 remaining patients (90.3% on antihypertensive medication), no significant differences were observed in Kt/V urea, UFV or percentage of weight gain from dry weight before HD between the standard HD and HD with BV-UFC groups (Tables 2 and 3).

**Table 2:** Patients’ general characteristics.

	Characteristics at study initiation
Number of patients (men/women)	31 (8/23)
Age (years)	68.0 ± 10.1
Group (A/B), *n*/*n*	14/17
Disease, *n*	
Diabetes mellitus	20
Chronic glomerulonephritis	6
Nephrosclerosis	4
ADPKD	2
Others	1
Comorbidities, *n*/*N*	
Cardiovascular disease	20/31
Cerebrovascular disease	6/31
Medication, *n* (%)	
Antihypertensive medicines	28 (90.3)
Renin-angiotensin system blocker	14 (45.2)
Calcium channel blocker	24 (77.4)
Beta blocker	8 (25.8)
Angiotensin receptor neprilysin inhibitor	5 (16.1)
Vitamin D analog	18 (58.1)
Erythropoiesis-stimulating agent	27 (77.4)
Hypoxia Inducible Factor Prolyl Hydroxylase	3 (9.7)
Laboratory findings	
Hb (g/dL)	11.4 ± 0.9
BUN (mg/dL)	54.47 ± 10.6
Serum creatinine (mg/dL)	10.1 ± 2.1
Na (mEq/L)	139.8 ± 3.3
K (mEq/L)	4.7 ± 0.6
Cl (mEq/L)	105.0 ± 3.4
Ca (mg/dL)	8.5 ± 0.6
Total protein (g/dL)	6.6 ± 0.6
Serum albumin (g/dL)	3.7 ± 0.3

This table shows the baseline characteristics of study participants, including gender, age, primary disease (diabetes mellitus, chronic glomerulonephritis, nephrosclerosis, ADPKD, others), comorbidities (cardiovascular disease, cerebrovascular disease) and medications (antihypertensive drugs, renin–angiotensin system blockers, calcium channel blockers, etc.). Additionally, laboratory findings such as Kt/V urea, blood urea nitrogen, and serum creatinine values for both standard HD and HD with BV-UFC are compared.

**Table 3:** HD characteristics.


HD					
HD duration (years)	8.5 ± 5.2				
Dry weight (kg)	59.2 ± 15.4				
HD times (min)	180	210	240	300	360
	6	4	18	2	1
Dialysate composition (mEq/L)	Na^+^		K^+^	Ca^++^	
	140		2.0	2.75	
Dialyzer type	CTA		PS	PAN AN69	
	25		5	1	
Dialyzer membrane surface area (m^2^)	2.42 ± 0.28				
HD/HDF	23/8				
	Standard HD		HD with BV-UFC	*P*-value	
*n* (total number of HD sessions)	186		186		
Treatment times (min)	231.6 ± 2.8		231.8 ± 2.8	.82	
Kt/V urea	1.51 ± 0.47		1.49 ± 0.042	.12	
Pre-dialysis weight gain percentage (%)	3.09 ± 0.12		3.17 ± 0.10	.29	
Ultrafiltration volume (L)	2.08 ± 0.13		2.11 ± 0.11	.61	
Mean ultrafiltration rate (mL/hr)	540.6 ± 14.5		548.1 ± 12.3	.47	
Blood flow (mL/min)	247.1 ± 1.9		246.9 ± 2.0	.82	
Pre-dialysis systolic BP (mmHg)	157.6 ± 1.2		154.3 ± 1.6	.01	
Post-dialysis systolic BP (mmHg)	148.6 ± 1.5		147.9 ± 1.6	.62	

This table presents detailed characteristics of the HD sessions conducted in this study, including session durations, types of dialyzers used, and treatment parameters for both standard HD and HD with BV-UFC.

Dialyzer type: CTA, cellulose triacetate; PS, polysulfone; PAN AN69, polyacrylonitrile AN69.

### Primary outcome

**Figure 3:**
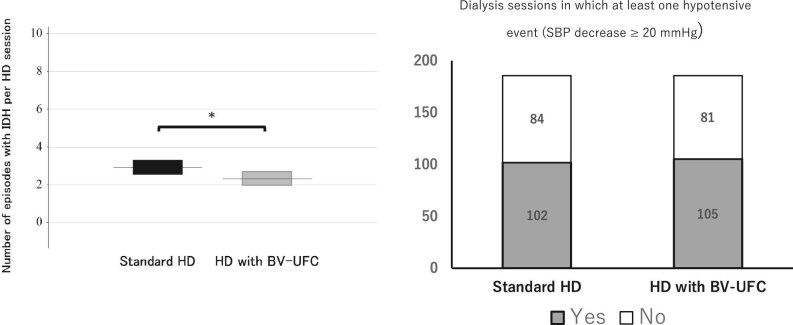
Primary outcomes. The figure compares the number of episodes of IDH per HD session between standard HD and HD with BV-UFC during different phases of the dialysis session and the number of dialysis sessions which at least one hypotensive event (systolic blood pressure decrease more than 20 mmHg), illustrating the effects of BV-UFC on hemodynamics. Bars indicate average ± SE. ^*^P < .05, ^**^P < .01.

The number of episodes with IHD per HD session was significantly different, 2.91 ± 0.38 episodes in standard HD compared with 2.32 ± 0.36 episodes in HD with BV-UFC (95% CI –1.08 to 1.04; *P* = .019). However, when evaluating the occurrence of IDH based on whether at least one event of systolic BP decrease ≥20 mmHg occurred within a session, no significant difference was observed between standard HD (102/186 sessions, 54.8%) and HD with BV-UFC (105/186 sessions, 56.5%) (*P* = .835) (Fig. 3).

### Secondary outcomes

The average PRR (mL/kg/h) over the entire HD session was significantly higher in HD with BV-UFC than in standard HD (95% CI 0.07–0.75; *P* = .019). The average PRR during the 0–1 h and 1–2 h after the initiation of HD was higher in HD with BV-UFC than in standard HD (95% CI 0.43 to 1.37; *P* < .001) (95% CI 1.04–2.07; *P* < .001). There was no significant difference in the average PRR during 2–3 h after initiating HD between the two groups (95% CI –0.34 to 0.83; *P* = .40). The average PRR during the 3–4 h after the initiation of HD was significantly lower in HD with BV-UFC than in standard HD (95% CI 0.53–1.86; *P* = .001).

**Figure 4:**
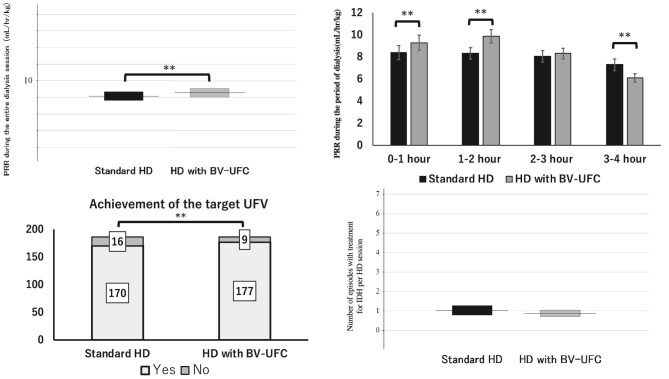
Secondary outcomes. This figure depicts PRR, achieving the target UFV and treatment for IDH per HD session, comparing standard HD and HD with BV-UFC. Bars indicate the average ± SE. ^*^P < .05.

The target UFV was not achieved in 16 of the 186 standard HD sessions or in 9 of the 186 HD sessions in HD with BV-UFC. The achievement rate of the target UFV was significantly higher in the HD with BV-UFC group than that in the standard HD group (*P* < .001). There was no significant difference between the two groups in the number of treatment episodes for IDH (95% CI –0.62 to –0.30; *P* = .46) (Fig. 4).

### UF rate, PRR and ΔBV during HD

**Figure 5:**
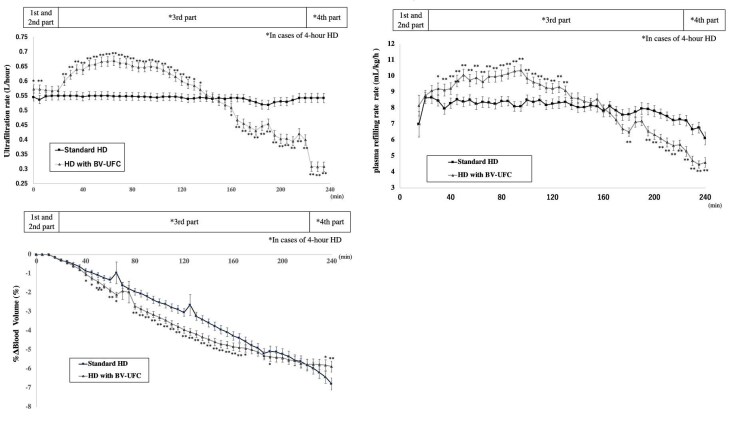
Changes in UF rate, PRR and ΔBV during HD sessions. This figure shows the phases of UF rate, PRR and ΔBV changes during HD sessions, including a constant UF rate period, a higher UF rate period, an automatic UF rate adjustment period and an automatic constant UF rate period. PRR values are expressed as 5-min averages (mL/kg/h), representing the mean plasma volume replenishment rate over successive time intervals. Bars indicate the average ± SE. ^*^P < .05, ^**^P < .01.

Between 25 and 135 min, the UF rates with BV-UFC were considerably higher than those with standard HD. However, after 160 min, the UF rate was notably lower. The PRR was significantly higher in HD with BV-UFC compared with standard HD during the period from 30 to 130 min. Conversely, PRR was significantly lower in HD with BV-UFC than in standard HD at 180 min and during the period from 195 to 240 min. %ΔBV was significantly lower in HD with BV-UFC compared with standard HD during the periods from 40 to 165 min, 80 to 170 min, and at 190 min. Conversely, %ΔBV was significantly higher in HD with BV-UFC than in standard HD during the period from 235 to 240 min (Fig. 5).

### BP and heart rate variation and %ΔBV at the end of HD

**Figure 6:**
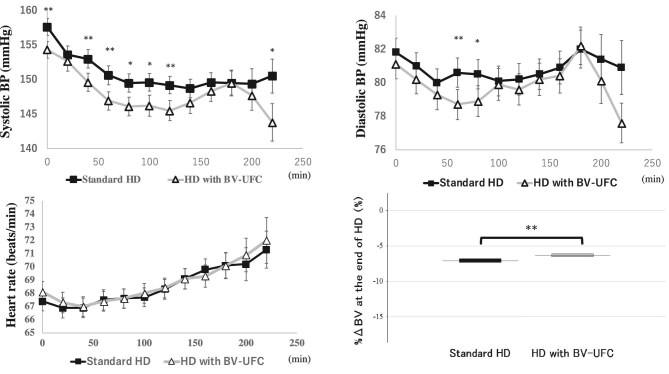
BP and heart rate variation and %ΔBV at the end of HD. This figure illustrates the variations in systolic BP, diastolic BP, heart rate and %ΔBV at the end of HD between standard HD and HD with BV-UFC. Bars indicate the average ± SE. ^*^P < .05, ^**^P < .01.

Systolic BP at the start of HD was significantly lower in HD with BV-UFC than that in standard HD (95% CI 0.93 to –5.71; *P* = .007). Furthermore, systolic BP from the start of HD up to 120 min (excluding the 20-min value) and 220 min after the start of HD was significantly lower in HD with BV-UFC than in standard HD. Diastolic BP was significantly lower in the HD with BV-UFC group than in the standard HD group at 60 and 80 min after the start of HD. No significant differences in heart rate were observed between the two groups throughout the HD period. At the end of HD, %ΔBV was –6.35 ± 0.21% (mean ± SE) in HD with BV-UFC and –7.09 ± 0.26% in standard HD. Thus, %ΔBV at the end of HD was significantly higher in HD with BV-UFC compared with the standard HD (95% CI 0.35 to 1.12; *P* < .001) (Fig. 6).

## DISCUSSION

In this study, we extended the third phase of HD with BV-UFC beyond default settings to examine its hemodynamic effects. It is possible that the number of IDH episodes is reduced due to the use of automatic UF adjustments. Additionally, BV-UFC improved target UFV achievement compared with standard HD. To our knowledge, this is the first study to show IDH reduction through BV-based UF regulation.

Currently, there is no universally accepted definition for IDH. According to the Kidney Disease Outcomes Quality Initiative (KDOQI) and European Best Practice Guidelines, IDH is defined as a decrease in systolic BP of ≥20 mmHg or a decrease in MAP of 10 mmHg accompanied by clinical symptoms requiring nursing interventions [21, 22]. Such a reduction in systolic BP of ≥20 mmHg or MAP of ≥10 mmHg has been reported in 68.9%–77.7% of cases [3, 2^3^]. Recently, IDH was often defined as “a systolic BP of less than 90 mmHg during a session” and “a nadir of less than 100 mmHg or <90 or 100 mmHg if the initial systolic BP was <160 mmHg or >160 mmHg, respectively,” as these definitions are associated with increased patient mortality [24, 25]. However, episodes meeting these definitions were extremely rare in our study population, with only 3 occurrences in 186 standard HD sessions and 1 occurrence in 186 BV-UFC sessions for the former definition, and 3 occurrences in standard sessions and 2 in BV-UFC-guided sessions for the latter. This rarity is likely due to the selection of outpatient dialysis patients with relatively stable overall conditions, who are less prone to severe hemodynamic instability.

Several factors contribute to the development of IDH, including excessive UF rates, chronic heart failure and inappropriate dry weight settings [1, 26]. Notably, both IDH and BP variability during HD considerably impact life expectancy, and an increase in the incidence of cardiovascular events in HD patients [2–4] IDH is prevalent among HD patients and represents a critical issue in HD treatment with substantial influences on patient prognosis. Effective strategies for the prevention of IDH include the use of hemodiafiltration (HDF) [27, 28], intermittent infusion hemodiafiltration [29], high-volume hemodiafiltration [30], cooler dialysate temperature [31, 31] and vasopressors, such as midodrine [32, 33], have demonstrated efficacy in reducing IDH episodes.

Modern HD devices, such as the one used in this study [Nikkiso Co., Ltd (equipped with a BV-UFC system)] [20] are capable of noninvasive BV monitoring with high accuracy by optically measuring variations in hematocrit, the primary blood component that determines total protein concentration [14–16, 34]. The system automatically adjusts the UF rate every 2 min based on real-time BV measurements from a built-in BV monitor. If %ΔBV falls below the target curve, the UF rate is reduced; if it drops more than 3% below, UF is temporarily suspended. Conversely, if %ΔBV exceeds the target, the UF rate is increased above the constant rate. This constant rate is calculated as the remaining UF volume divided by the remaining HD time. Thus, the BV-UFC system makes precise UF adjustments based on BV, which are challenging to perform manually.

To date, only one study has compared the utility of BV-UFC with standard HD [20]. This crossover trial was conducted to compare standard HD and HD with BV-UFC. Similar to the present study, a considerable difference in ΔBV was observed at the end of dialysis. However, no substantial differences were found in the frequency of IDH or achievement rate of the target UFV. We hypothesized that the BV-UFC system in its default settings would likely have a minimal impact on IDH frequency and therefore focused on PRR as a more direct measure of the system's effectiveness in optimizing plasma refilling dynamics. However, adjustments made during the preparatory phase revealed unexpected benefits in IDH reduction, leading to the revision of the primary endpoint. This highlights the importance of dynamic assessment during preparatory phases and suggests further exploration of BV-UFC settings in clinical practice.

In this study, we compared mean PRR during HD between standard HD and HD with BV-UFC to assess hemodynamic changes. Although mean UFV per session was similar in both groups, mean PRR was higher in the BV-UFC group throughout HD and especially in the first 2 h. This increase is likely due to the BV-UFC's automatic UF rate increase during the early phase when excess fluid volume is higher [35]. Changing the settings from the default configuration to those used in this study unexpectedly resulted in a lower frequency of IDH in the HD with BV-UFC compared with standard HD (Fig. 3), contrary to our initial expectations and prior study. This difference may be due to the shortened second period (10 min instead of 60 min) and the extended third period in our study, allowing earlier automatic UF adjustments based on BV and PRR. In the previous study, a higher UF rate in the prolonged second period led to greater drops in systolic BP and %ΔBV, likely impacting hemodynamics. Here, reducing the UF rate in the second part and extending the third part enabled better plasma refilling, leading to improved intravascular volume maintenance and a lower frequency of IDH. Notably, UFV was similar between groups, but plasma refilling was significantly greater in the BV-UFC group. In the BV-UFC group, the target UFV was achieved even with the UF rate reduced to 50% during the fourth phase. For patients with sufficient UF progress, the target UFV or a value very close to it was typically reached by the end of the third phase. Consequently, the remaining UFV during the fourth phase was minimal, allowing participants to achieve a dry weight within ±0.3 kg of the target in the majority of sessions.

In our study, we evaluated IDH occurrence using two different approaches: (i) the proportion of sessions with at least one IDH event (systolic BP decrease ≥20 mmHg) and (ii) the number of IDH episodes per session. While no significant difference was observed in the proportion of IDH sessions between standard HD and HD with BV-UFC (54.8% vs 56.5%, *P* = .8353), the number of IDH episodes per session was significantly lower in the BV-UFC group (2.91 ± 0.38 vs 2.32 ± 0.36, *P* = .019). These findings suggest that BV-UFC may not completely prevent IDH, but it could reduce its recurrence within a session, contributing to improved hemodynamic stability. Previous studies have also highlighted the importance of evaluating IDH frequency rather than session-based IDH occurrence alone. Studies by Rootjes *et al*. (2022), Bullen *et al*. (2019) and Chesterton *et al*. (2009) demonstrated that interventions such as high-volume hemodiafiltration and cool dialysate effectively reduce the overall frequency of IDH episodes [30, 36, 37]. These findings support the hypothesis that strategies focusing on minimizing IDH recurrence may enhance hemodynamic stability during dialysis. This distinction between session-based IDH occurrence and IDH frequency may be crucial in assessing the clinical relevance of BV-UFC and other interventions. Further studies are needed to explore whether a reduction in IDH recurrence per session translates to improved long-term outcomes for HD patients.

The higher %ΔBV observed at the end of HD in the BV-UFC group can be attributed to the system's dynamic adjustment of UF rates. By reducing the UF rate during the later phases of dialysis, particularly when the amount of excess body water was low, the BV-UFC system effectively maintained hemodynamic stability while minimizing the risk of excessive fluid removal. These findings highlight the potential advantages of BV-UFC in preventing hemodynamic complications during HD.

In the present study, systolic BP was significantly lower in HD with BV-UFC than in standard HD from the start of sessions. This result differs from those of a previous study [20]. Extending the duration of HD and lowering the UF rate during HD can reduce the incidence of IDH and improve control of BP [13, 38]. It has been also reported that shorter dialysis durations with higher UF rates are associated with increased BP and a higher frequency of IDH [28]. A high UF rate may be associated with sympathetic nervous system activation, renin–angiotensin system activation, endothelial stiffness [39]. In this study, entering the third stage earlier likely prevented excessive UF in the early phase of HD. Reducing the UF rate in the later phase may lessen physiological stress and improve BP control. If BV-UFC has similar BP benefits as extended HD sessions, it could enhance the long-term prognosis for HD patients.

Recently, Nikkiso's HEMOMASTER system combines BV monitoring and feedback controls. While both systems of BV-UFC and HEMOMASTER system are developed by Nikkiso and are part of their dialysis equipment, they differ in functionality and design. HEMOMASTER system calculates the ΔBV decrease rate per liter of UF, and healthcare providers select from five target lines ranging from gentle to steep. Gentler lines have been associated with fewer adverse effects such as IDH, sweating and fatigue [40]. Similar to BV-UFC, HEMOMASTER features a constant UF rate period, but lacks a higher UF rate period. Incorporating gentler target lines, as suggested by HEMOMASTER, may have further reduced IDH in this study.

The findings of this study suggest that BV-UFC may contribute to improved hemodynamic stability during hemodialysis by reducing the recurrence of IDH episodes. Although the proportion of dialysis sessions with at least one IDH event did not differ significantly between groups, BV-UFC significantly reduced the number of IDH episodes per session. This indicates that BV-UFC may help mitigate recurrent hypotensive episodes rather than completely preventing IDH occurrence. Further research is needed to determine whether this reduction in IDH frequency translates into improved patient outcomes, such as cardiovascular protection and better long-term fluid management. Future studies should also assess the efficacy of BV-UFC in populations at higher risk of IDH, such as hospitalized patients or those with compromised cardiovascular function.

The study has several limitations. IDH was assessed only by systolic BP or MAP reductions, excluding clinical symptoms. This study has the exclusion of subjective clinical symptoms, such as sweating and fatigue, from the analysis. While these symptoms were observed during dialysis sessions, they were not systematically recorded or analyzed due to their subjective nature. Instead, the study focused on objective data that could be quantified and reliably measured, such as the frequency of IDH and BP changes. One limitation of this study is the evaluation of IDH incidence using BP measurements at 20-min intervals, which may underestimate the actual frequency of IDH due to missed episodes between measurements. This limitation is consistent with findings from Chaara *et al*. [41], which emphasize the need for more frequent BP monitoring to accurately capture IDH events. Hospitalized patients with fluctuating dry weight were not included, and the short-term crossover design did not assess long-term outcomes. Home BP monitoring was also lacking, despite lower systolic BP at the start of HD with BV-UFC. Future studies should address these gaps to better evaluate BV-UFC's effectiveness. This study also did not evaluate the effectiveness of BV-UFC under conditions such as HD with a cold dialysate temperature (e.g. 35.5°C) or high-volume hemodiafiltration (total convective volume >23 L/session). These conditions may influence plasma refilling dynamics and UF outcomes, and further studies are needed to validate the efficacy of BV-UFC in these settings.

This study shows that adjusting the UF rate automatically based on BV reduces IDH frequency while achieving similar UFV as standard HD within the same treatment time. It is the first to report that automatic UF adjustment can reduce IDH frequency while maintaining safety compared with standard HD.

## Supplementary Material

sfaf141_Supplemental_File

## Data Availability

The data that support the findings of this study are available from the corresponding author (S.H.), upon reasonable request.

## References

[1] Kanbay M, Ertuglu LA, Afsar B et al. An update review of intradialytic hypotension: concept, risk factors, clinical implications and management. Clin Kidney J 2020;13:981–93. 10.1093/ckj/sfaa078 33391741 PMC7769545

[2] Tislér A, Akócsi K, Borbás B et al. The effect of frequent or occasional dialysis-associated hypotension on survival of patients on maintenance haemodialysis. Nephrol Dial Transplant 2003;18:2601–5. 10.1093/ndt/gfg450 14605284

[3] Flythe JE, Xue H, Lynch KE et al. Association of mortality risk with various definitions of intradialytic hypotension. J Am Soc Nephrol 2015;26:724–34. 10.1681/ASN.2014020222 25270068 PMC4341481

[4] Liu Q, Wang W, Wu X et al. Intra-dialytic blood pressure variability is a greater predictor of cardiovascular events in hemodialysis patients. BMC Nephrol 2023;24:1–9. 10.1186/s12882-023-03162-w 37101121 PMC10134565

[5] Raine AEG. The susceptible patient. Nephrol Dial Transplant 1996;11:6–10. 10.1093/ndt/11.supp2.6. 10.1093/ndt/16.8.1657 8803986

[6] Sato M, Horigome I, Chiba S et al. Autonomic insufficiency as a factor contributing to dialysis-induced hypotension. Nephrol Dial Transplant 2001;16:1657–62. 10.1093/ndt/11.supp2.11 11477170

[7] Leunissen KML, Kooman JP, Van Kuijk W et al. Preventing haemodynamic instability in patients at risk for intra-dialytic hypotension. Nephrol Dial Transplant 1996;11:11–15. 10.1111/j.1523-1755.2004.00881.x 8803987

[8] Flythe JE, Kimmel SE, Brunelli SM. Rapid fluid removal during dialysis is associated with cardiovascular morbidity and mortality. Kidney Int 2011;79:250–7. 10.1038/ki.2010.383 20927040 PMC3091945

[9] Pillon L, Piccoli A, Lowrie EG et al. Vector length as a proxy for the adequacy of ultrafiltration in hemodialysis. Kidney Int 2004;66:1266–71. 10.1111/j.1523-1755.2004.00881.x 15327426

[10] Termorshuizen F, Dekker FW, Van Manen JG et al. Relative contribution of residual renal function and different measures of adequacy to survival in hemodialysis patients: an analysis of the Netherlands Cooperative Study on the Adequacy of dialysis (NECOSAD)-2. J Am Soc Nephrol 2004;15:1061–70. 10.1097/01.ASN.0000117976.29592.93 15034110

[11] Daugirdas JT. Pathophysiology of dialysis hypotension: an update. Am J Kidney Dis 2001;38:S11–7. 10.1053/ajkd.2001.28090 11602456

[12] Schroeder KL, Sallustio JE, Ross EA. Continuous haematocrit monitoring during intradialytic hypotension: precipitous decline in plasma refill rates. Nephrol Dial Transplant 2004;19:652–6. 10.1093/ndt/gfg590 14767022

[13] Saran R, Bragg-Gresham JL, Levin NW et al. Longer treatment time and slower ultrafiltration in hemodialysis: associations with reduced mortality in the DOPPS. Kidney Int 2006;69:1222–8. 10.1038/sj.ki.5000186 16609686

[14] Mancini E, Santoro A, Spongano M et al. Continuous on-line optical absorbance recording of blood volume changes during hemodialysis. Artif. Organs 1993;17:691–4. 10.1111/j.1525-1594.1993.tb00616.x 8215949

[15] Yoshida I, Ando K, Ando Y et al. A new device to monitor blood volume in hemodialysis patients. Ther Apher Dial 2010;14:560–5. 10.1111/j.1744-9987.2010.00845.x 21118363

[16] Dong Z, Fuentes LR, Rao S et al. Closed loop ultrafiltration feedback control in hemodialysis: a narrative review. Toxins (Basel) 2024;16:1–18. 10.3390/toxins16080351 PMC11360213 39195761

[17] Kolben Y, Gork I, Peled D et al. Continuous monitoring of advanced hemodynamic parameters during hemodialysis demonstrated early variations in patients experiencing intradialytic hypotension. Biomedicines 2024;12:1177. 10.3390/biomedicines12061177 38927384 PMC11200556

[18] Jongejan M, Gelinck A, van Geloven N et al. Effect of absolute blood volume measurement–guided fluid management on the incidence of intradialytic hypotension-associated events: a randomised controlled trial. Clin Kidney J 2024;17:1–10. 10.1093/ckj/sfae128 38774440 PMC11106788

[19] Reddan DN, Szczech LA, Hasselblad V et al. Intradialytic blood volume monitoring in ambulatory hemodialysis patients: a randomized trial. J Am Soc Nephrol 2005;16:2162–9. 10.1681/ASN.2004121053 15930095

[20] Ookawara S, Ito K, Uchida T et al. Hemodialysis crossover study using a relative blood volume change-guided ultrafiltration control compared with standard hemodialysis: the BV-UFC study. Ren Replace Ther 2020;6:1–10. 10.1186/s41100-020-00295-8

[21] K/DOQI Workgroup . K/DOQI clinical practice guidelines for cardiovascular disease in dialysis patients. Am J Kidney Dis 2005;45:S1–153. 15806502

[22] Kooman J, Basci A, Pizzarelli F et al. EBPG guideline on haemodynamic instability. Nephrol Dial Transplant 2007;22:22–44. 10.1093/ndt/gfm019 17507425

[23] Kuipers J, Oosterhuis JK, Krijnen WP et al. Prevalence of intradialytic hypotension, clinical symptoms and nursing interventions—A three-months, prospective study of 3818 haemodialysis sessions dialysis and transplantation. BMC Nephrol 2016;17:1–11. 10.1186/s12882-016-0231-9 26922795 PMC4769826

[24] Flythe JE, Xue H, Lynch KE et al. Association of mortality risk with various definitions of intradialytic hypotension. J Am Soc Nephrol 2015;26:724–34. 10.1681/ASN.2014020222 25270068 PMC4341481

[25] Chou JA, Streja E, Nguyen DV et al. Intradialytic hypotension, blood pressure changes and mortality risk in incident hemodialysis patients. Nephrol Dial Transplant 2018;33:149–59. 10.1093/ndt/gfx037 28444336 PMC5837776

[26] Brennan JM, Ronan A, Goonewardena S et al. Handcarried ultrasound measurement of the inferior vena cava for assessment of intravascular volume status in the outpatient hemodialysis clinic. Clin J Am Soc Nephrol 2006;1:749–53. 10.2215/CJN.00310106 17699282

[27] Donauer J, Schweiger C, Rumberger B et al. Reduction of hypotensive side effects during online-haemodiafiltration and low temperature haemodialysis. Nephrol Dial Transplant 2003;18:1616–22. 10.1093/ndt/gfg206 12897103

[28] Locatelli F, Altieri P, Andrulli S et al. Hemofiltration and hemodiafiltration reduce intradialytic hypotension in ESRD. J Am Soc Nephrol 2010;21:1798–807. 10.1681/ASN.2010030280 20813866 PMC3013537

[29] Koda Y, Aoike I, Hasegawa S et al. Feasibility of intermittent back-filtrate infusion hemodiafiltration to reduce intradialytic hypotension in patients with cardiovascular instability: a pilot study. Clin Exp Nephrol 2017;21:324–32. 10.1007/s10157-016-1270-z 27125432 PMC5388713

[30] Rootjes PA, Chaara S, de Roij van Zuijdewijn CLM et al. High-volume hemodiafiltration and cool hemodialysis have a beneficial effect on intradialytic hemodynamics: a randomized cross-over trial of four intermittent dialysis strategies. Kidney Int Rep 2022;7:1980–90. 10.1016/j.ekir.2022.06.021 36090495 PMC9459077

[31] Zoccali C, Tripepi G, Neri L et al. Effectiveness of cold HD for the prevention of HD hypotension and mortality in the general HD population. Nephrol Dial Transplant 2023;38:1700–6. 10.1093/ndt/gfad003 36649682

[32] Prakash S, Garg AX, Heidenheim AP et al. Midodrine appears to be safe and effective for dialysis-induced hypotension: a systematic review. Nephrol Dial Transplant 2004;19:2553–8. 10.1093/ndt/gfh420 15280522

[33] Brunelli SM, Cohen DE, Marlowe G et al. The impact of midodrine on outcomes in patients with intradialytic hypotension. Am J Nephrol 2018;48:381–8. 10.1159/000494806 30423552

[34] Johner C, Chamney PW, Schneditz D et al. Evaluation of an ultrasonic blood volume monitor. Nephrol Dial Transplant 1998;13:2098–103. 10.1093/ndt/13.8.2098 9719173

[35] Mitsides N, Pietribiasi M, Waniewski J et al. Transcapillary refilling rate and its determinants during haemodialysis with standard and high ultrafiltration rates. Am J Nephrol 2019;50:133–43. 10.1159/000501407 31288231

[36] Bullen A, Rifkin D, Trzebinska D. Individualized cool dialysate as an effective therapy for intradialytic hypotension and hemodialysis patients’ perception. Ther Apher Dial 2019;23:145–52. 10.1111/1744-9987.12761 30226300 PMC6422756

[37] Chesterton LJ, Selby NM, Burton JO et al. Cool dialysate reduces asymptomatic intradialytic hypotension and increases baroreflex variability. Hemodial Int 2009;13:189–96. 10.1111/j.1542-4758.2009.00355.x 19432693

[38] Tandon T, Sinha AD, Agarwal R. Shorter delivered dialysis times associate with a higher and more difficult to treat blood pressure. Nephrol Dial Transplant 2013;28:1562–8. 10.1093/ndt/gfs597 23348881 PMC3685306

[39] Kim IS, Kim S, Yoo TH et al. Diagnosis and treatment of hypertension in dialysis patients: a systematic review. Clin Hypertens 2023;29:1–12. 10.1186/s40885-023-00240-x 37653470 PMC10472689

[40] Xu Z, Liang G, Wang Y et al. Effectiveness and safety of HAEMOMASTER system in hemodialysis patients. Altern Ther Health Med 2023;29. 38064620

[41] Chaara S, Rootjes P, Bergtop M et al. #672 Intradialytic hypotension and adverse symptoms are not related. Nephrol Dial Transplant 2024;39:gfae069–e0760–672. 10.1093/ndt/gfae069.760

