# Supplementary material for: Effectiveness of blood volume change–guided ultrafiltration control (BV-UFC) in hemodialysis: a crossover comparative study
Source: Clin Kidney J. 2025 May 6;18(5):sfaf141. doi: 10.1093/ckj/sfaf141 (PMC12092915; doi:10.1093/ckj/sfaf141)
Supplement: sfaf141_Supplemental_File [file sfaf141_supplemental_file.docx]

# SUPPLEMENTARY MATERIALS

Figure S1: The frequency at which systolic BP decreased by more than 20%–30% relative to baseline BP

This figure depicts the number of episodes of IDH per HD session, a systolic BP drop of 20% or more per HD session, 30% or more per HD session, comparing standard HD and HD with BV-UFC. Bars indicate the average ± SE.

Number of episodes with a systolic BP

drop of 20% or more per HD session


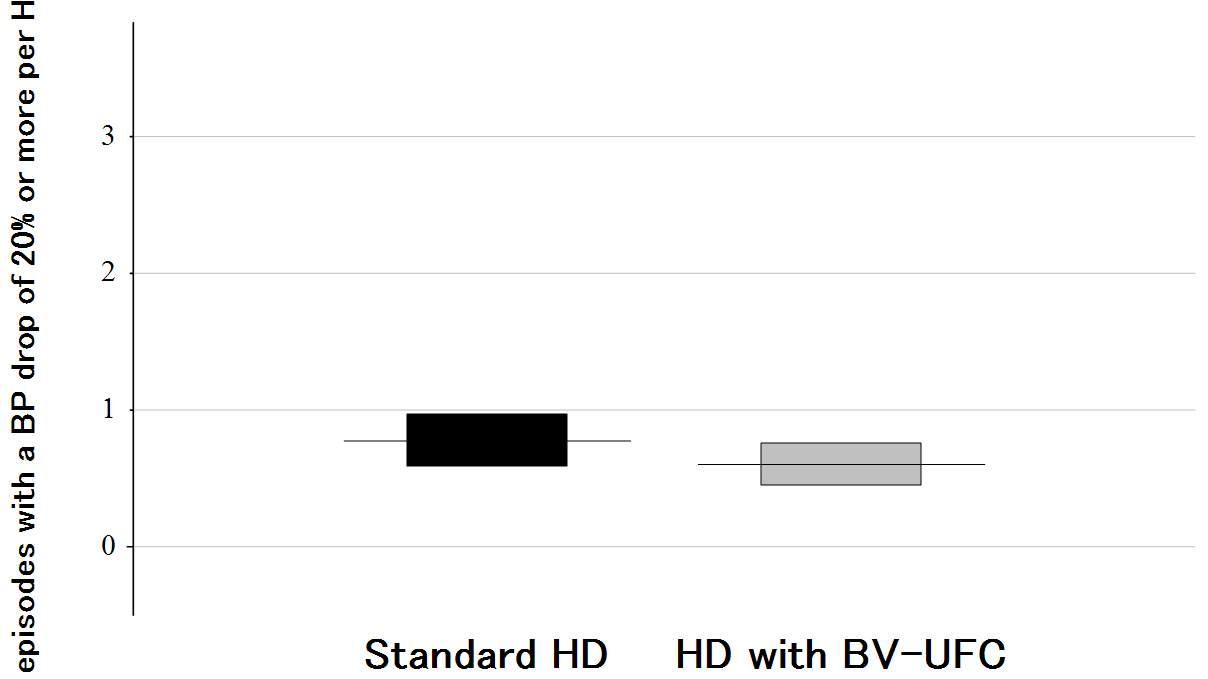


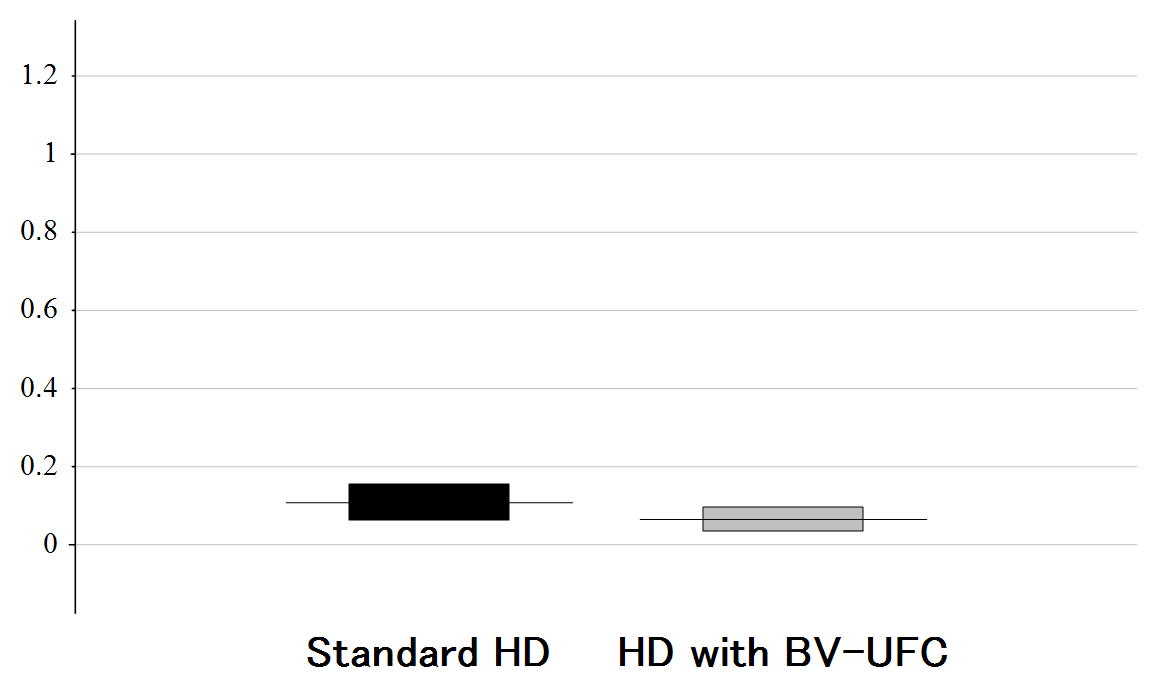


Number of episodes with a systolic BP

drop of 30% or more per HD session

There were no significant differences in the frequency of occurrences in which systolic BP decreased by more than 20%-30% between both groups (95% CI, -0.46 to 0.12; p = 0.24) (95% CI, -0.12 to 0.03; p = 0.26).

Figure S2: Standard deviation(SD) and coefficient of variation(CV)

The figure shows a comparison of SD and CV between standard HD and HD with BV-UFC. Bars indicate the average ± SE.


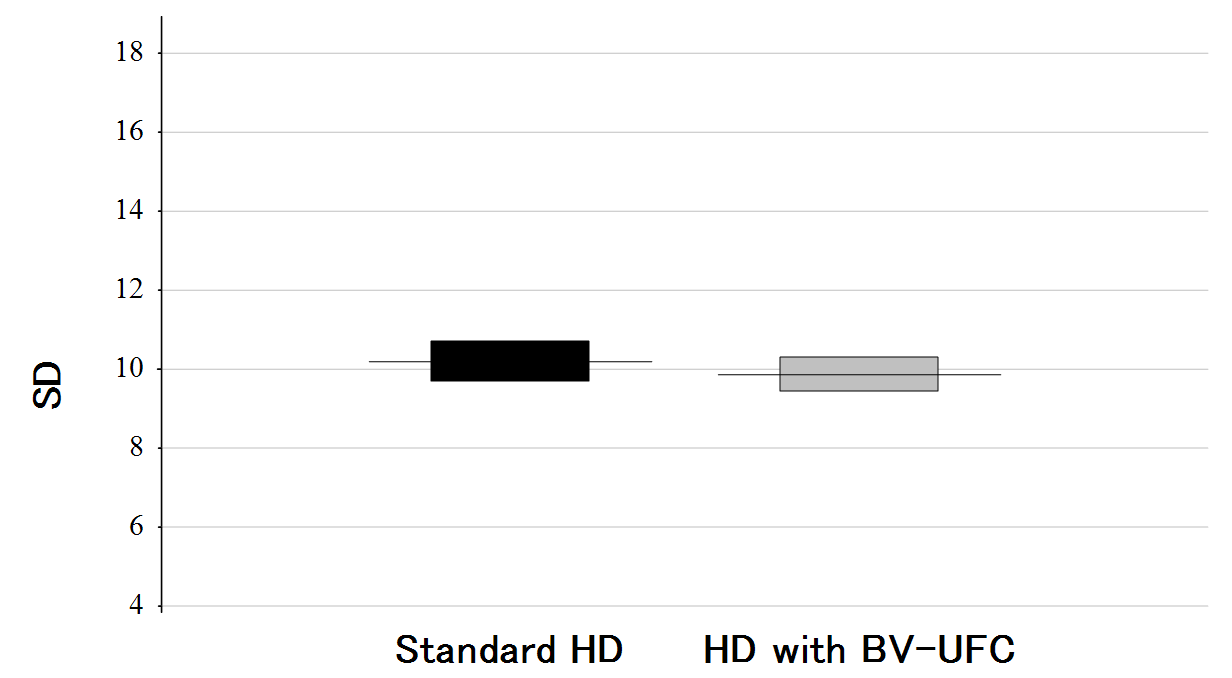


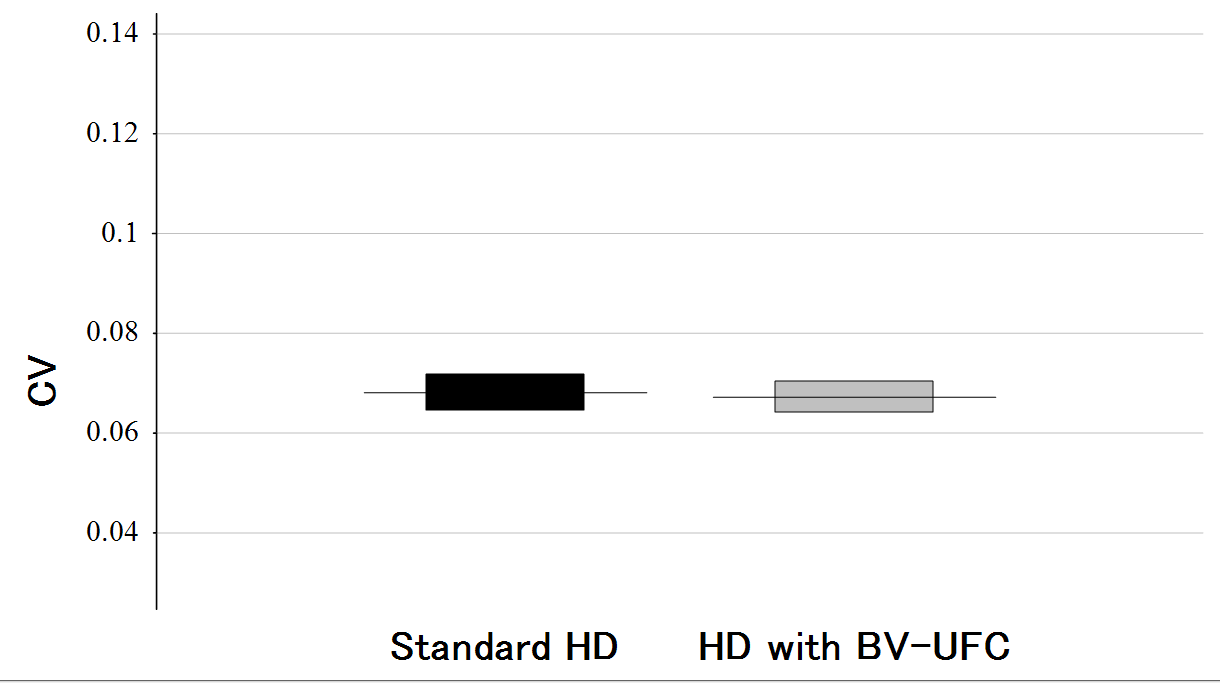


No significant differences were observed in the SD and CV per HD session between the two groups (95% CI, -0.27 to 0.93; p = 0.27) (95% CI, -0.003 to -0.004; p = 0.66).
